# Examinations of Bilateral Epileptiform Activities in Hippocampal Slices Obtained From Young Mice

**DOI:** 10.3389/fncel.2020.593840

**Published:** 2021-01-20

**Authors:** Haiyu Liu, Peter L. Carlen, Liang Zhang

**Affiliations:** ^1^Department of Neurosurgery, The First Hospital of Jilin University, Changchun, China; ^2^Krembil Research Institute, University Health Network, Toronto, ON, Canada; ^3^Department of Medicine (Neurology), University of Toronto, Toronto, ON, Canada; ^4^Department of Physiology, University of Toronto, Toronto, ON, Canada

**Keywords:** CA3, dorsal hippocampal commissure, epilepsy, *in vitro*, mouse, seizures

## Abstract

Bilateral interconnections through the hippocampal commissure play important roles in synchronizing or spreading hippocampal seizure activities. Intact hippocampi or bilateral hippocampal slices have been isolated from neonatal or immature rats (6–7 or 12–21 days old, respectively) and the mechanisms underlying the bilateral synchrony of hippocampal epileptiform activities have been investigated. However, the feasibility of examining bilateral epileptiform activities of more developed hippocampal circuitry *in vitro* remains to be explored. For this, we prepared bilateral hippocampal slices from C57 black mice, a strain commonly used in neuroscience and for genetic/molecular modifications. Young mice (21–24-day-old) were used in most experiments. A 600-μm-thick slice was obtained from each mouse by horizontal vibratome sectioning. Bilateral dorsal hippocampal and connecting dorsal hippocampal commissure (DHC) tissues were preserved in the slice and extrahippocampal tissues were removed. Slices were recorded in a submerged chamber mainly at a room temperature (21–22°C). Bilateral CA3 areas were monitored by extracellular recordings, and unilateral electrical stimulation was used to elicit CA3 synaptic field potentials. The unilateral stimulation could elicit population spikes in the contralateral CA3 area. These contralateral spikes were attenuated by inhibiting synaptic transmission with cobalt-containing medium and were abolished when a cut was made at the DHC. Self-sustained and bilaterally correlated epileptiform potentials were observed following application of 4-aminopyradine and became independent after the DHC cut. Bilateral hippocampal activities were detectable in some slices of adult mice and/or at 35–36°C, but with smaller amplitudes and variable waveforms compared to those observed from slices of young mice and at the room temperature. Together, these observations suggested that examining bilateral epileptiform activities in hippocampal slices of young mice is feasible. The weaknesses and limitations of this preparation and our experimentation are discussed.

## Introduction

The rodent hippocampus has strong bilateral connections through the hippocampal commissure (Amaral and Lavenex, 2007). Bilateral hippocampal interconnections play important roles in cognitive functions (Jordan, 2020) and in synchronizing or spreading seizure activities. Intrahippocampal electroencephalographic (EEG) recordings are commonly used to assess bilateral hippocampal activities during physiological behaviors (Suzuki and Smith, 1987; Buzsáki et al., 2003) and in models of epileptic seizures (Wang et al., 2014; Li et al., 2018). However, it is difficult to isolate bilateral activities mediated by the hippocampal commissure *in vivo* because the hippocampus has extensive connections with other brain structures and extrahippocampal input signals greatly influence hippocampal EEG activities.

The bilateral epileptiform activities of the rat hippocampus have been investigated *in vitro*. Specifically, Khalilov et al. (1997, 1999, 2003) isolated intact hippocampi (with or without septum) with their connecting dorsal hippocampal commissures (DHCs) from 6 to 7-day-old rats. The isolated hippocampi and DHCs were monitored in a chamber comprising three independent compartments in which each hippocampus and its DHC were independently perfused with artificial cerebrospinal fluid (ACSF). Synchronous population epileptiform activities were observed from bilateral CA3 areas by unilateral or bilateral hippocampal exposure to ACSF containing a convulsive agent such as high K^+^, 4-aminopyradine (4-AP), or kainic acid. Application of the sodium channel blocker tetrodotoxin to the DHC or administration of high Mg^2+^ or the glutamate NMDA receptor antagonist AP5 to the convulsant-exposed hippocampus abolished or greatly decreased these synchronous epileptiform activities. Subsequently, Durand and co-workers prepared bilateral hippocampal slices from immature 12 to 21-day-old rats (Toprani and Durand, 2013a,b; Wang et al., 2014). These slices, 350 or 750 μm thick, were obtained by vibratome sectioning, and each slice preserved bilateral ventral hippocampal-entorhinal areas and connecting ventral hippocampal commissure (VHC) tissues. Synchronous epileptiform field potentials were observed from bilateral CA3/CA1 areas following perfusion of the slices with 4-AP-containing ACSF. These synchronous epileptiform activities were suppressed or greatly attenuated when a cut was made at the VHC or by low-frequency electrical stimulation of the VHC. The latter effects were mediated by GABAb receptor-dependent mechanisms. Together, these findings indicate that the DHC and VHC have critical roles in the synchronization of bilateral hippocampal epileptic activities. However, the intact hippocampi or bilateral hippocampal slices were largely obtained from neonatal or immature rats, and the feasibility of *in vitro* examination of bilateral epileptiform activities in more developed hippocampal circuitry remains to be further explored.

Mouse models have been increasingly employed for neurobiology research largely due to advances in genetic/molecular manipulation methods. The corpus callosum and hippocampal commissures were found to have inheritable defects in some inbred mouse strains (Schimanski et al., 2002; MacPherson et al., 2008; Bohlen et al., 2012) or were poorly developed in some transgenic models (Barallobre et al., 2000; Shu et al., 2003). To date, no study has examined bilateral hippocampal activities in a mouse model *in vitro*.

Here, we explored whether brain slices obtained from young mice are suitable for examining bilateral hippocampal epileptiform activities *in vitro*. We prepared slices from C57 black mice as this strain is widely employed in neuroscience research and genetic/molecular manipulations as a background strain. Young mice at 21–24 days of age were used in most experiments as proof-of-principle approach to explore the feasibility of this slice preparation. Mice of 28–32 days or 9–13 months old were used in some experiments. The rodent hippocampus resides under the cerebral cortex and is orientated from the lateral–ventral to the medial–dorsal part of the brain such that the dorsal hippocampus and connecting DHC is more compact and closer to the brain midline relative to the ventral hippocampus and connecting VHC. Consequently, we focused on dorsal hippocampal and connecting DHC tissues for convenience in slice preparation and consistency in the preservation of bilateral hippocampal activities *in vitro*.

## Materials and Equipment

Animals: Male C57 black mice (C57BL/6N, Charles River Laboratory, Saint-Constant, Quebec, Canada).

Chemicals and pharmacological agents: sodium chloride, potassium chloride, sodium monophosphate, magnesium sulfate, calcium chloride, sodium bicarbonate, D-glucose, sucrose, and 4-aminopyrideine (Sigma-Aldrich, Oakville. Ontario, Canada).

Vibratome: Leica VT1200 (Leica Biosystems Inc., Ontario, Canada).

Recording amplifier: Multiclamp 700A (Molecular Devices; Sunnyvale, California, USA).

Digitizer: Digidata 1550 (Molecular Devices; Sunnyvale, California, USA).

Electrophysiology software: Pclamp 10 (Molecular Devices; Sunnyvale, California, USA).

Stimulator: Grass S88 (Grass Medical Instruments, Warwick, Rhode Island, USA).

Glass tubes for making recording electrodes: thin wall with filaments (World Precision Instruments, Sarasota, Florida, USA).

Micromanipulators: Hydraulic WR-6, Narishige International USA Inc., Amityville, New York, USA).

Wires for making stimulation electrodes: Polyamide-insulated stainless-steel wires (110 μm outer diameter; Plastics One, Ranoake, Virginia, USA).

Dissecting scope: Stereo binocular dissecting scope (Nikon Instruments Inc., Melville, New York, USA).

Surgical blades and handle: Scalpel blade #11 and handle #4 (Fine Science Tools, North Vancouver, British Columbia, Canada).

Fine painting brush: D'Artisan shoppe miniature brushes #01 (https://www.artnews.com/art-news/product-recommendations/best-detail-paint-brushes-1202687045/).

Submerged recording chamber and slice holding frame: constructed by our lab (see details in Wu et al., 2002, 2005).

Statistical software: Sigmaplot (Systat Software Inc., San Jose, California, USA) and Origin (Northampton, Massachusetts, USA).

Carbogen gas: 95% O_2_/5% CO_2_ (Praxair Canada Inc, Mississauga, Ontario, Canada).

## Methods

### Preparation of Bilateral Dorsal Hippocampal Slices

All the experiments conducted in this study were reviewed and approved by the Animal Care Committee of the University Health Network in accordance with the Guidelines of the Canadian Council on Animal Care.

Male C57 black mice of 21–24 days old were used in most experiments. Mice of ages 28–32 days-old or 9–12 months-old were used in some experiments. Each mouse was anesthetized by an intraperitoneal injection of sodium pentobarbital (100 mg/kg) and underwent a trans cardiac infusion with cold, dissection ACSF before decapitation. The brain was quickly dissected out and placed in oxygenated ice-cold dissection ACSF for approximately 2 min for further cooling.

**Figure 1:**
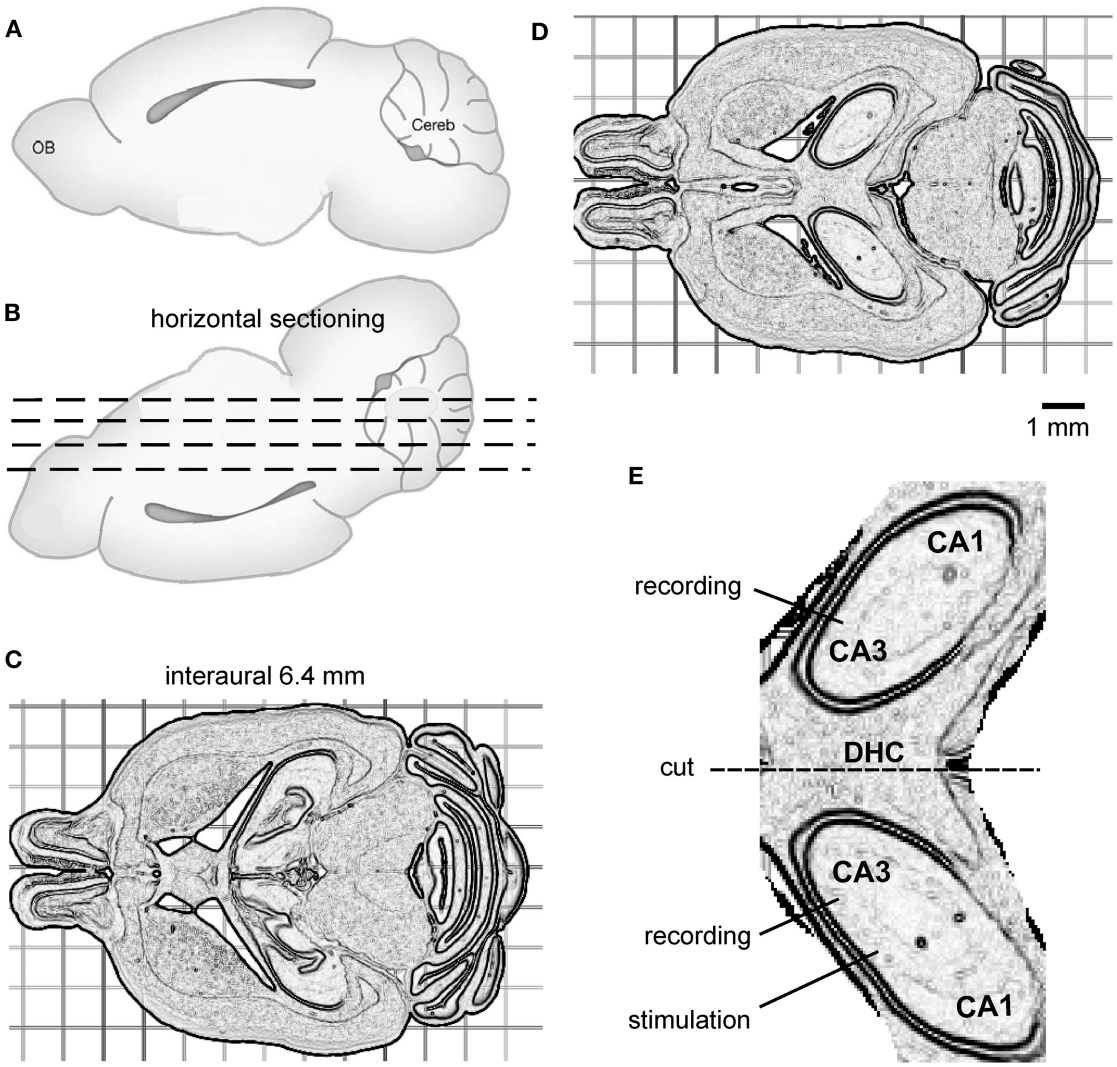
The preparation of bilateral hippocampal slices. **(A,B)** Cartoons showing a mouse brain with its cerebral cortex or dorsal surface facing down for horizontal vibratome sectioning (dashed lines). **(C)** Several horizontal sections were cut from the ventral part of the brain to reach the horizontal level corresponding to interaural 6.4 mm. **(D)** A 600-μm-thick slice was then obtained. The slice encompassed bilateral dorsal hippocampal and connecting DHC tissues. **(E)** An enlarged drawing showing isolated, bilaterally connected hippocampal tissue. The general positions of a stimulating electrode and two recording electrodes are illustrated.

For vibratome sectioning, the brain was glued onto a metal plate with its dorsal surface facing down and the frontal part aligning the horizontal cut plane (Figures 1A,B). Horizontal vibratome sectioning, as illustrated by the dashed lines in Figure 1B, was performed in ice-cold dissection only ACSF. Several 300–500-μm-thick sections were obtained from the ventral part of the brain to reach a horizontal level corresponding to interaural 6.4 mm (Figure 1C). This level was visually determined during vibratome section as per the brain atlas of C57 black mice (Rosen et al., 2000; Williams, 2000) and the relevant histological images available at http://www.mbl.org/about_us/about.php. Then, a 600-μm-thick slice was obtained (Figure 1D). Bilateral dorsal hippocampal and connecting DHC tissues were preserved in this slice and hippocampal areas were loosely connected, or often detached, from cortical tissues (Figure 1D). In addition, hippocampal areas were separated from striatum tissues by lateral ventricles (Figure 1D). As such, the bilateral hippocampal and connecting DHC tissues could easily be isolated from surrounding cortical and striatal tissues. Using this protocol, one slice with preservation of bilateral hippocampal tissues and the connecting DHC could be obtained from each mouse, whereas slices obtained at other horizontal planes could encompass bilateral hippocampal tissues but without the connecting midline DHC tissue.

To isolate bilateral hippocampal and connecting DHC tissues, the 600-μm-thick slice was transferred to a glass dish containing ice-cold dissection ACSF using a suction pipette. Extrahippocampal tissues were removed using a surgical blade. If needed, residual dentate gyrus tissue was gently detached from the hippocampus using a fine brush. A schematic illustration of the bilateral dorsal hippocampal slice is shown in Figure 1E. After being isolated, the bilateral hippocampal slice was transferred to a glass beaker containing oxygenated (5% CO_2_/95% O_2_) standard ACSF for ≥1 h before recordings.

The ACSF used for dissection comprised 280 mM sucrose, 3.5 mM KCl, 0.5 mM CaCl_2_, 6 mM MgCl_2_, 10 mM HEPES, and 10 mM D-glucose (pH 7.35–7.40). The standard ACSF comprised 125 mM NaCl, 3.5 mM KCl, 1.25 mM NaH_2_PO_4_, 2 mM CaCl_2_, 1.3 mM MgSO_4_, 25 mM NaHCO_3_, and 10 mM D-glucose (pH 7.35–7.40 when aerated with 95% O_2_/5% CO_2_). To induce epileptiform activities, 4-AP was added to the standard ACSF at a concentration of 100 μM.

### Recording Apparatus

A custom submerged chamber was used for *in vitro* recordings (Wu et al., 2002, 2005). The recording chamber has inner dimensions of 3.5 × 5 × 20 mm (depth × width × length). The bilateral hippocampal slice was held on a stainless-steel fine mesh (0.015-in grid length) via a frame made of fine stainless-steel wires. The mesh was set 1.5 mm above the bottom of the chamber to allow the perfusion of the oxygenated ACSF to both sides of the slice. The oxygenated ACSF, kept at a room temperature of 21–22°C or warmed to 35–36°C (see below), was perfused to the slice at ~15 ml/min. During the perfusion, the slice was kept at a minimal submerged level to achieve an effective exchange between the oxygenated ACSF and the slice tissue. A water bath underneath the recording chamber was set at 21°C or 35°C via an automatic temperature control unit, and humidified gas of 95% O_2_/5% CO_2_ could pass over the perfusate to increase local oxygen tension in the recording chamber.

To perfuse slices with warmed ACSF, aerated (95% O_2_/5% CO_2_) ACSF was warmed to 35–36°C using a large warm bath with automatic temperature control. Warmed ACSF was added into a perfusion bottle, which was surrounded by an automatic heating blanket to keep ACSF at 35–36°C. Under these conditions, the perfusate temperature, measured by a fine temperature probe near the perfused slice, was near (≤0.5°C) ACSF temperature in the perfusion bottle (Wu et al., 2005).

### Extracellular Recordings of Bilateral Hippocampal Slices

The bilateral hippocampal slice was placed in the recording chamber with its stratum alveus layer (axons of pyramidal neurons) facing down or stratum radiatum layer (apical dendrites of pyramidal neurons) facing up for convenience in placement of stimulating and recording electrodes. The slice was trans illuminated by a fiber-optic light source and visualized under a dissection microscope. A stainless-steel wire net was placed onto the striatum radiatum layer (apical dendrites of pyramidal neurons) of the slice to secure its placement but without disrupting tissue integrity. During recording, the slice was perfused by oxygenated (5% CO_2_/95% O_2_) ACSF at a high rate (~15 mL per min). Both the top and bottom surfaces of the slice were exposed to perfusate. Humidified gas with 95% O_2_/5% CO_2_ was passed over the perfusate to increase local oxygen tension (Wu et al., 2002, 2005). Recordings were performed at a perfusate temperature of 21–22°C or 35–36°C. Previous work from our and other labs has shown that fast, both-side perfusion of rodent brain slices is important to maintain spontaneous population activities under submerged recording conditions (Wu et al., 2002, 2005; Hájos and Mody, 2009).

Extracellular recordings by glass electrodes were used to monitor local field potentials. The recording electrodes were filled with a solution containing 150 mM NaCl and 2 mM HEPES (pH 7.4; resistance of 1~2 MΩ). Two recording electrodes were positioned by micromanipulators in the cell body layer of bilateral CA3 areas identified as a narrow, yellowish band near the edge of the CA3 area (Figure 1E). A two-channel amplifier was used to sample local field potentials. The amplifier was set with an input frequency band of 0 to 1,000 or 3,000 Hz and an amplification gain of 50–100. The output signals of the amplifier were digitized at 5,000–10,000 Hz. pClamp software was used for data acquisition, storage, and analyses. A twisted wire bipolar electrode was used for local afferent stimulation. The stimulating electrode was positioned by a coarse manipulator in the left or right CA3 area near the recording electrode (Figure 1E). The stimulation site was distal to the midline relative to the ipsilateral recording site. We used this approach to elicit local synaptic field potentials and assess the spreading of synaptic signals from the stimulated site to the contralateral site through the DHC. Constant current pulses (0.1-ms duration, at a near-maximal intensity of 150 μA) were generated by a Grass stimulator and delivered every 20 s through a photoelectric isolation unit. To disconnect bilateral hippocampal tissues, a cut was made at the DHC region using a surgical scalpel (Figure 1E). The recording and stimulating electrodes were withdrawn before the cut, and subsequently repositioned.

### Data Analysis

*In vitro* epileptiform field potentials were recognized according to the recommendations of the International League Against Epilepsy and American Epilepsy Society Joint Translational Task Force (Raimondo et al., 2017; Dulla et al., 2018). Briefly, ictal-like discharges were considered as repeated spike waveforms with peak amplitudes of ≥1 mV, durations (spike-expressing components of ≥10 s, and mean interevent intervals of ≥90 s. Interictal spike like potentials were recognized as intermittent or periodic events with peak amplitudes of ≥0.5 mV and durations (components with low-amplitude spikes) of ≤1 s. Single- or multi-unit spike activities that displayed base duration of ≤5 ms, variable amplitudes and spikes rates were not considered as epileptiform field potentials because these spikes activities were not uniformly detected in the targeted area.

The peak amplitudes of evoked CA3 population spikes were measured from averages of 5–8 consecutive responses. Cross-correlation analysis was performed using Origin software (Northampton, MA, USA). Original data collected in a wide frequency band (0 to 1,000 or 3,000 Hz) were treated with a band-pass filter (2–500 Hz, Bessel, 8 pole) to remove slow drifts and high-frequency noise before cross-correlation analysis. Interictal-like field potentials were recognized as intermittent events with peak amplitudes of ≥0.5 mV and durations (components with low-amplitude spikes) of ≤1 s. An event detection function (threshold search method) of pClamp was used to automatically detect interictal field potentials. If needed, original data were also treated with a high-pass filter (2 Hz) before the event detection. Detected events were visually inspected and artifacts were excluded. The onset time lags of ictal- and interictal-like field potentials were measured from original wide-band signals.

Statistical analysis was conducted using Sigmaplot or Origin software. Data were presented as means and standard error of the mean (SEM) throughout the text. A Student's *t*-test or Mann–Whitney *U* test was used to compare maximum correlation of bilateral ictal signals or interevent intervals measured before and after the DHC was cut. Statistical significance was set at *p* < 0.05.

## Results

In our present experiments, one bilateral hippocampal slice was obtained from each mouse. The number of bilateral hippocampal slices examined are indicated below for convenience in data presentation. Slices that did not encompass bilateral hippocampal tissues and the connecting midline DHC are not included in following data presentation except where specified.

### Observations From Bilateral Hippocampal Slices of Young Mice

#### Unilaterally Elicited and Bilaterally Recorded CA3 Population Spikes

After initial practice, the preparation of bilateral hippocampal slices was carried out in 22 mice of 21–24 days-old (one slice per mouse; see Section “Methods”). Slices (0.6 mm thickness) with visually appreciable preservation of bilateral hippocampal tissues and the connecting DHC were obtained from 15 of the 22 mice used. In slices from remaining seven mice, the midline DHC tissue was evidently truncated or disrupted under visual inspection. This was due to vibratome sectioning of the 0.6 mm slice at an undesired horizontal plane or dissection-related tissue damage. No recording was made from the seven slices as our focus was on bilaterally connected dorsal hippocampal activities.

**Figure 2:**
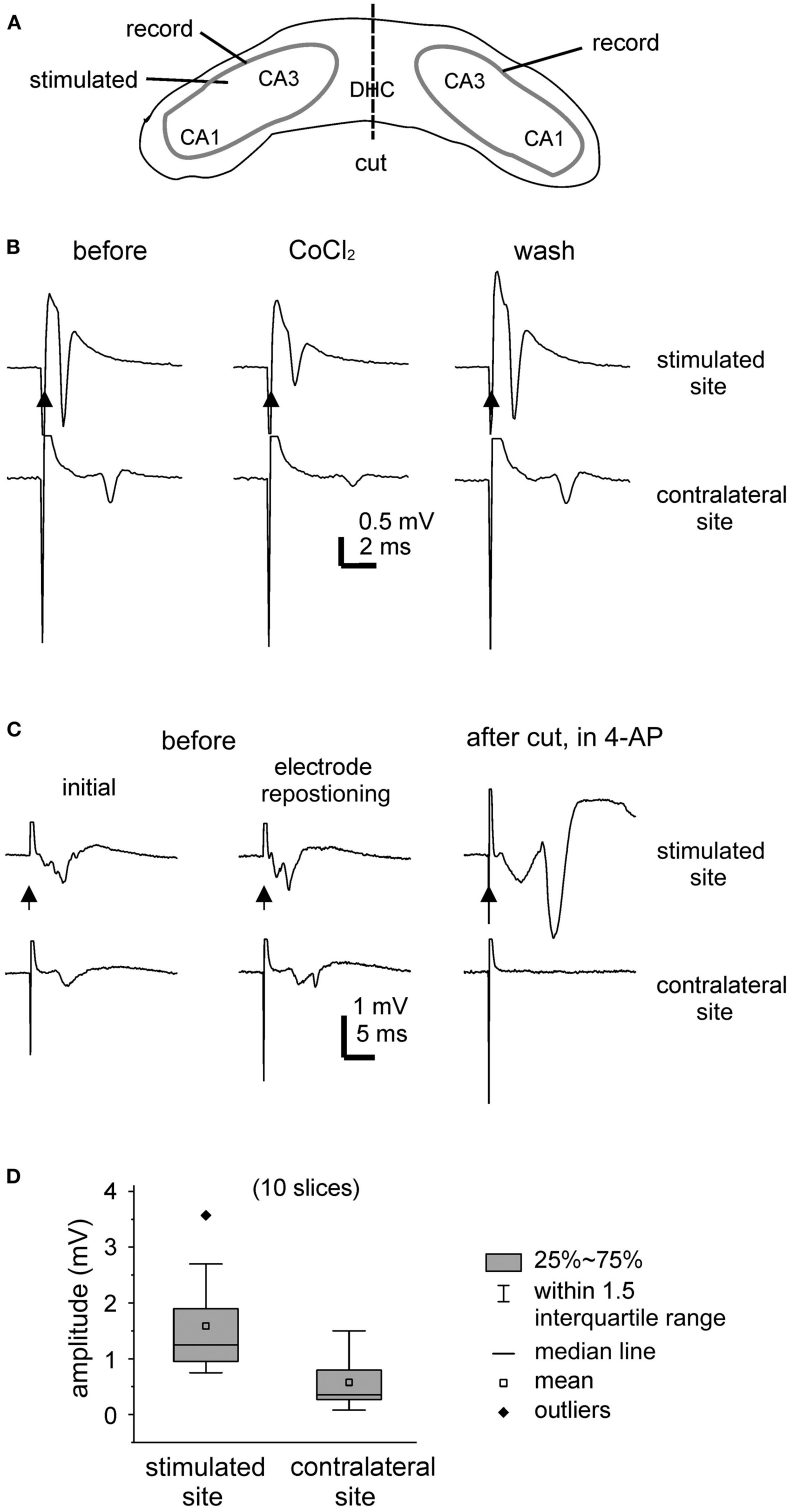
Unilaterally elicited and bilaterally recorded synaptic field potentials. **(A)** A carton showing the positions of stimulating and recording electrodes. **(B,C)** Bilateral hippocampal slices were obtained from 2 mice (21- or 23-days-old). CA3 population spikes were evoked unilaterally and recorded bilaterally. Illustrated traces were averaged from five consecutive responses. Filled arrows denote the unilateral afferent stimulation. **(B)** The evoked responses collected before (left), after the application of 2 mM CoCl_2_ for about 8 min (middle), and after washing out the cobalt (right). **(C)** The evoked responses collected initially (left), after repositioning two recording electrodes (middle), and following the application of 100 μM 4-AP and after a cut that was made at the DHC (right). **(D)** Box plots showing the peak amplitudes of evoked CA3 population spikes. Measurements were made from the stimulated and contralateral sites in 10 slices.

Evoked field potentials were examined largely during slice perfusion with standard ACSF. Figure 2A is a schematic illustration of general position of stimulating and recording electrodes. Field potentials were evoked unilaterally and recorded bilaterally from CA3 somatic areas (Figures 2B,C). Evoked responses were readily detectable from the stimulated hippocampal tissues, with large amplitudes when recordings were made near the stimulating electrodes. Evoked responses recorded from the contralateral or unstimulated CA3 area were more evident when recordings were made toward the midline. Recording electrodes were often repositioned in individual slices to achieve large responses (Figure 2C).

Of the 15 slices with visually appreciable preservation of bilaterally connected hippocampal tissues mentioned above, unilaterally elicited and bilaterally recorded responses were observed in 10 slices (one slice per mouse). In response to the unilateral stimulation at near-maximal intensity (150 μA), the peak amplitudes of the CA3 population spikes were 1.6 ± 0.3 mV and 0.6 ± 0.2 mV in the stimulated site and contralateral site, respectively (hereafter means and SE, *n* = 10 slices; Figure 2D). The mean time lag between the peaks of corresponding bilateral spikes was 2.6 ± 0.3 ms. To inhibit synaptic transmission, a general calcium channel blocker CoCl_2_ was added to the standard ACSF (He et al., 2009). Both the stimulated and contralateral CA3 population spikes were reversibly attenuated by CoCl_2_ application (at 2 mM for 7–8 min, *n* = 2 slices; Figure 2B). As schematically presented in Figure 2A, a cut was made at the DHC to disconnect bilateral hippocampal issues. The DHC cut was perform in five bilateral hippocampal slices following the application of 4-AP (see below). Evoked CA3 population spikes were detectable in the stimulated site but not in the contralateral (unstimulated) site after the cut in all 5 slices examined (Figure 2C).

Evoked responses were observed from the stimulated site but not from the corresponding contralateral (unstimulated) site in 5 of the 15 slices with visually appreciable preservation of bilaterally connected hippocampal tissues mentioned above. The lack of detectable contralateral responses might be due partly to fine damages and/or functional deterioration of the DHC as direct stimulation of the contralateral site (by repositioning the stimulating electrode) elicited CA3 responses in two of the five slices.

**Figure 3:**
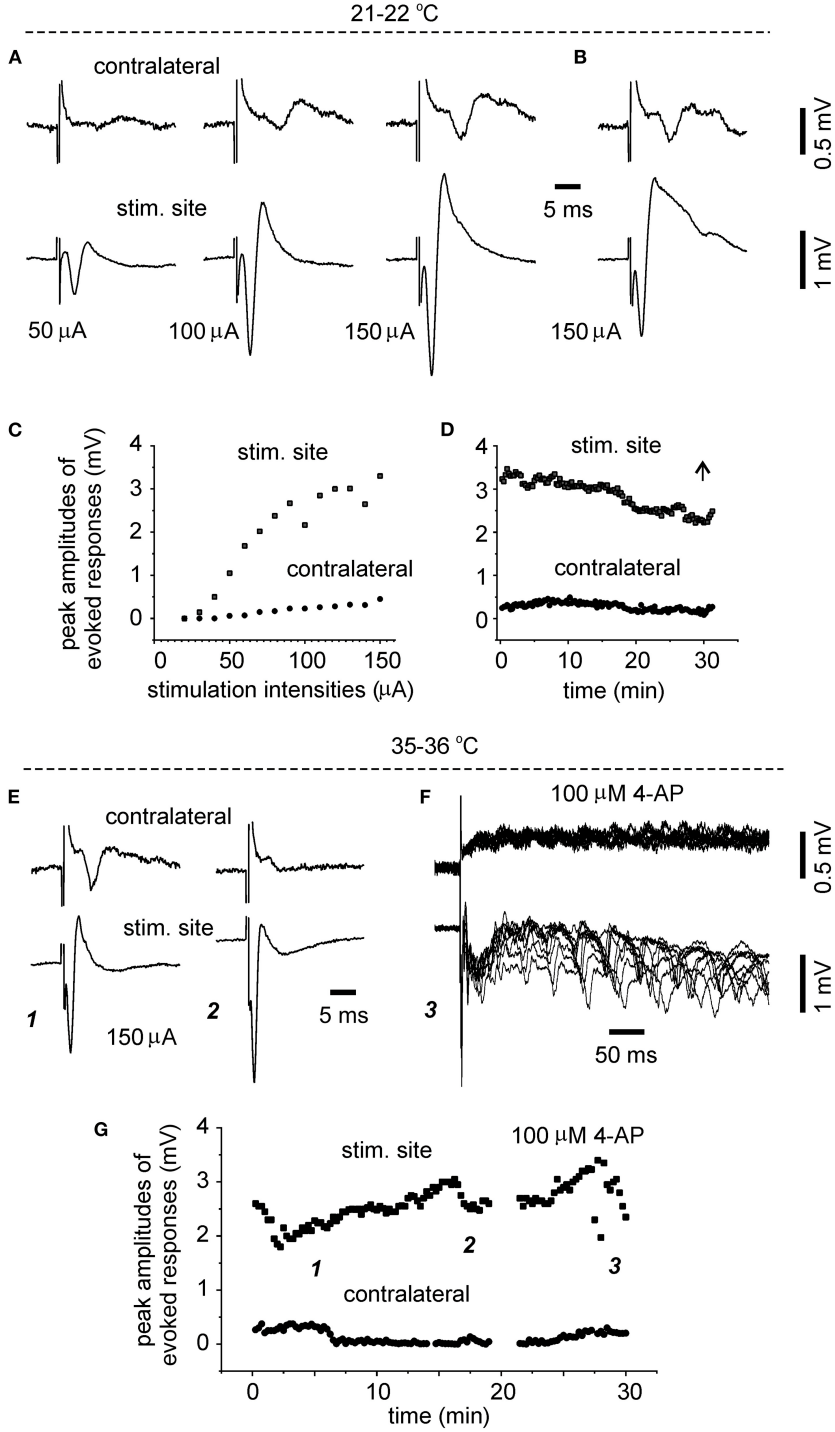
Time- and temperature-dependent changes in evoked bilateral hippocampal responses. A bilateral hippocampal slice was obtained from a 29-day-old mouse. Unilaterally elicited and bilaterally recorded synaptic field potentials were collected initially at a room temperature [21–22°C, **(A–D)**] and then at 35–36°C **(E–G)**. **(A,C)** Stimuli were applied every 15 s and at incremental intensities (20–150 μA, 10 μA per increment). Four consecutively evoked responses at each intensity were averaged. Selected averages were presented in **(A)**, and peak amplitudes of averaged responses were plotted vs. stimulation intensities in **(C)**. **(B,D)** Responses evoked by constant stimulations of 150 μA over a 30-min period. Peak amplitudes of each evoked responses were plotted vs. time in **(D)**. Averages from the last four consecutive responses were presented in **(B)**. **(E,G)** Responses evoked every 15 s by constant stimulations of 150 μA at 35–36°C. Peak amplitudes of each evoked responses were plotted vs. time in **(G)**. Four consecutive responses evoked at indicated times in **(G)** were presented in [**(E)** and **(F)**]. Superimposed traces in **(F)** presented 10 consecutive responses.

To explore whether evoked bilateral hippocampal responses can be observed in slices of more developed mice and at more physiological temperatures, bilateral hippocampal slices were similarly prepared from three mice of 28–32 days-old (one slice per mouse). Slices with visually appreciable preservation of bilateral hippocampal tissues and the connecting DHC were obtained from two mice but evoked bilateral CA3 responses were observed from one slice. We examined the input-output relation of evoked bilateral responses in this slice. In response to the stimulations with incremental intensities of 20–150 μA, the amplitudes of bilateral responses were gradually increased and plateaued in an intensity range of 100–150 μA (Figures 3A,C). While being evoked by constant stimulations and monitored at the room temperature over a 30-min period, bilateral responses showed slight decreases in amplitude and subtle changes in waveform (Figures 3B,D). In response to similar stimulations and monitored at 35–36°C in next 30 min, evoked responses of the stimulated site were increased in amplitudes and altered in waveform whereas contralateral responses were decreased (Figures 3E,G). Following application of 100 μM 4-AP at 35–36°C, evident epileptiform responses were elicited from the stimulated site, and low-amplitude slow responses were noticeable in the contralateral site (Figure 3F). Self-sustained epileptiform field potentials (not shown) were observed from the stimulated and contralateral sites. Together the above observations suggested that bilaterally connected dorsal hippocampal tissues can be preserved in slices of young mice. The CA3 population spikes recorded from the unstimulated sites might have resulted from the synaptic signal spreading from the stimulated site through the DHC. However, evoked contralateral responses were substantially decreased while being monitored at 35–36°C, suggesting that DHC-mediated bilateral connectivity may be unstable or deteriorated at more physiological temperatures (see Section “Discussion”).

#### Correlated Epileptiform Field Potentials Observed From Bilateral CA3 Areas

Epileptiform field potentials were induced by perfusing the slices with standard ACSF containing 100 μM 4-AP (Barbarosie and Avoli, 1997). Of the 15 slices from mice of 21–24 days-old and with visually appreciable preservation of bilaterally connected hippocampal tissues mentioned above, the effects of 4-AP application were examined in 10 slices from which evoked bilateral CA3 responses were observed (Figure 2); 4-AP was not applied to other five slices without evoked bilateral responses as our focus was on bilaterally correlated epileptiform activity. Self-sustained epileptiform field potentials were observed following 4-AP application in all the 10 slices tested. Specifically, ictal-like discharges with or without infrequent interictal-like events were observed from five slices; interictal-like field potentials but not ictal-like discharges were observed from other five slices.

#### Ictal Discharges Observe Before and Following the DHC Cut

**Figure 4:**
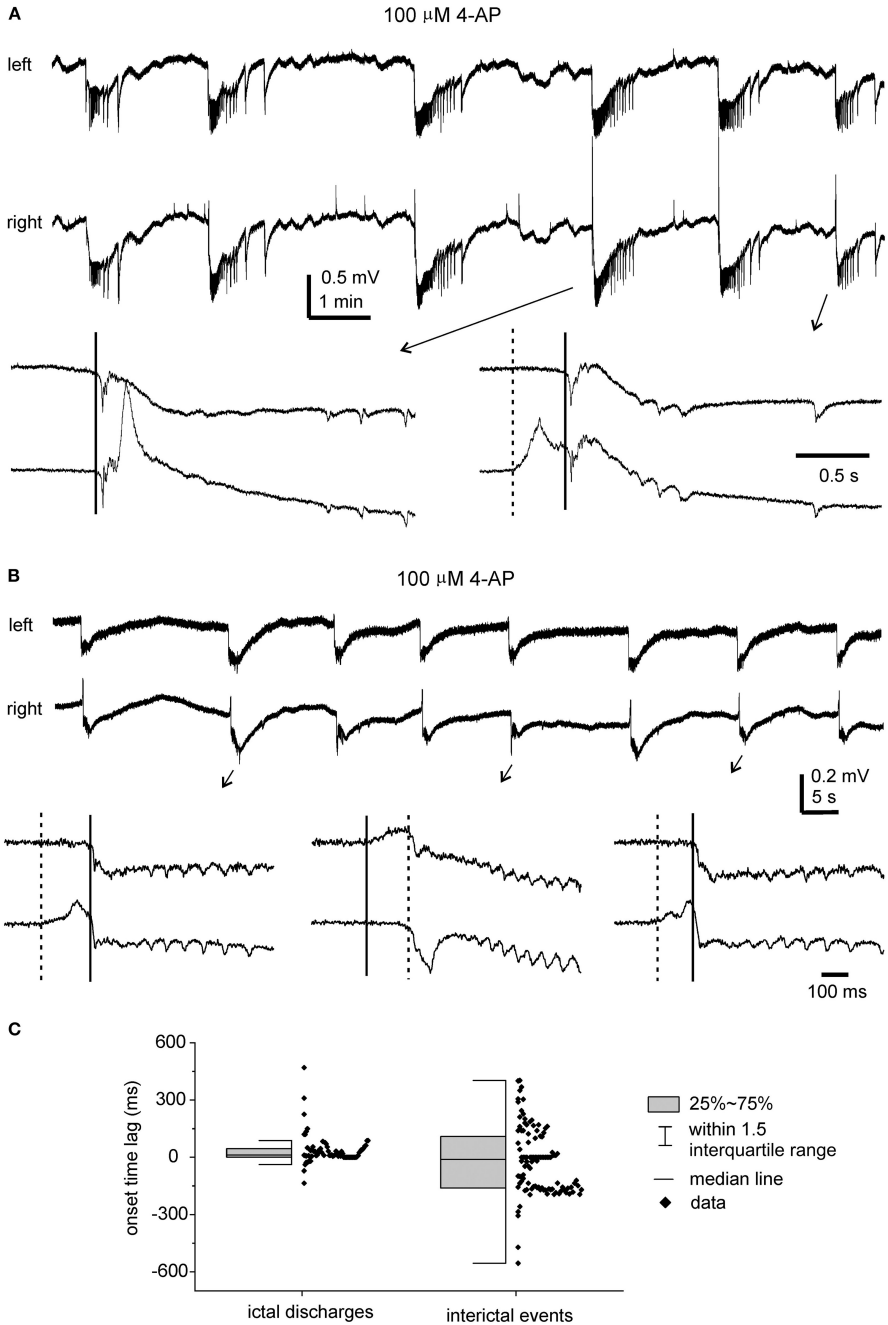
4-AP induced, bilaterally correlated epileptiform field potentials. Bilateral hippocampal slices were obtained from 2 mice (22- or 24-days-old). Epileptiform field potentials were induced by 100 μM 4-AP. **(A)** Recurrent ictal-like discharges illustrated after treatment with a low-pass filter (500 Hz, Bessel, 8-pole). Arrowed events were expanded to show initial parts of corresponding bilateral discharges. Solid and dotted lines denote putative onset times of right and left hippocampal discharges. **(B)** Intermittent interictal-like events were illustrated after treatment with a low-pass (200 Hz) filter. Arrowed events were expanded below and similarly illustrated as in **(A)**. **(C)** Measured onset time lags of bilaterally correlated ictal discharges (37 events from 5 slices) and interictal events (108 events from other 5 slices).

**Figure 5:**
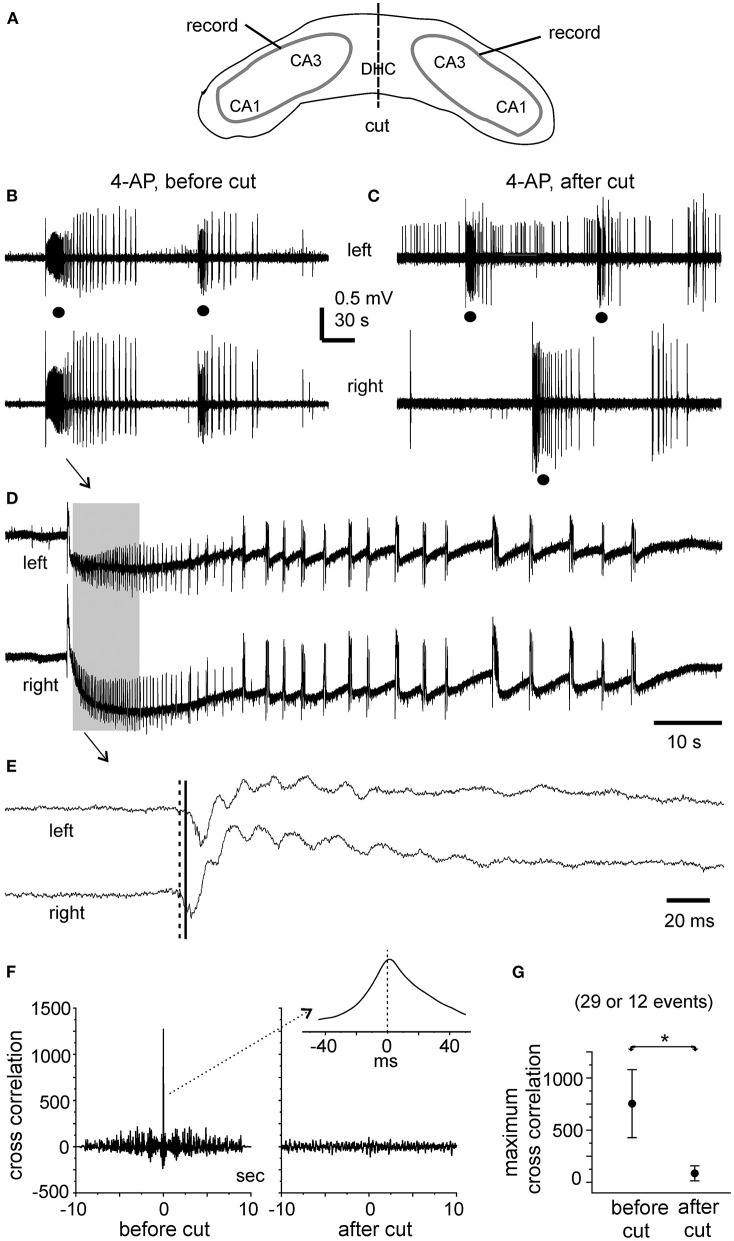
Bilateral ictal discharges observed before and following a cut at DHC. **(A)** A diagram showing the positions of the recording electrodes. **(B**–**E)** A bilateral hippocampal slice prepared from a 22-day-old mouse. Self-sustained ictal discharges (filled circles) induced by 100 μM 4-AP and illustrated after treatment with a band-pass filter (2–500 Hz). Arrowed ictal events in **(B)** were expanded in **(D)** and squared events in **(D)** were expanded in **(E)**. **(E)** Cross-correlation analysis performed for the first bilateral discharges illustrated in **(B)** (before the cut) and for the unilateral discharge and corresponding contralateral signals illustrated in **(C)** (after the cut). Ten-second data segments (denoted by the gray box in **(D)** were used to generate cross-correlation plots. **(F)** Maximum cross-correlation assessed for 29 bilaterally correlated discharges (5 intact slices) and for 12 unilateral discharges and associated contralateral signals (2 slices after the DHC was cut). Data in **(G)** are presented as means ± 95% confidence intervals. *before *vs*. after the cut, *p* = 0.012, Student's *t*-test.

In the five slices that displayed ictal discharges and monitored before the DHC cut, these ictal discharges appeared concurrently in bilateral CA3 areas but the onsets of individual corresponding discharge events, visually inspected as shown in Figures 4A, 5A,B,D,E were variable. In 37 events recorded from the five slices, the mean onset time lag of the corresponding bilateral ictal discharges was 60.9 ± 16.0 ms (Figure 4C, left). Specifically, corresponding bilateral discharges showed no evident time lag at onset (or zero time lag) in 12 events (32%); discharges of left site led (by 25–470 ms) or lagged (from 20 to 136 ms) contralateral discharges at onset in 15 (41%) or 10 (27%) events respectively. The durations of the left and right CA3 discharges were 29.7 ± 4.8 and 28.0 ± 4.3 s, respectively; the interevent interval of ictal discharges was 94.6 ± 15.3 s. Cross-correlation analysis was performed for the early parts of bilateral discharges (10 s long, denoted by a gray box in Figure 5D). Highly correlated signals were observed in all bilateral spike activities analyzed (Figures 5F,G).

After the DHC was cut, self-sustained ictal discharges remained detectable in two of the five slices; however, these discharges occurred independently in isolated left and right CA3 areas (Figure 5C) and showed no evident correlation (Figures 5F,G). The isolated left and right CA3 discharges were 23.4 ± 2.6 and 36.7 ± 7.3 s long and with interevent intervals of 157.1 ± 38.7 and 140.8 ± 15.8 s, respectively (*n* = 10 and 6 events). Compared to bilaterally correlated ictal discharges measured before the DHC cut (durations of 56.0 ± 9.9 s and interevent intervals of 160.6 ± 22.9 s, 12 events/2 slices), these isolated regional discharges were shorter in duration (Mann–Whitney *U* test, *p* < 0.05) but not significantly different in interevent intervals. In remaining three slices that were monitored for ≥15 min after the DHC cut, interictal field potentials with independent appearance in isolated right and left CA3 areas were observed in two slices (interevent intervals of 20.3 ± 1.8 and 23.3 ± 1.7 s, 36 and 29 events measured from 12. 5 min); no self-sustained epileptiform potential was observed from isolated left and right hippocampal areas in other slice.

#### Interictal Events Observed Before and Following the DHC Cut

**Figure 6:**
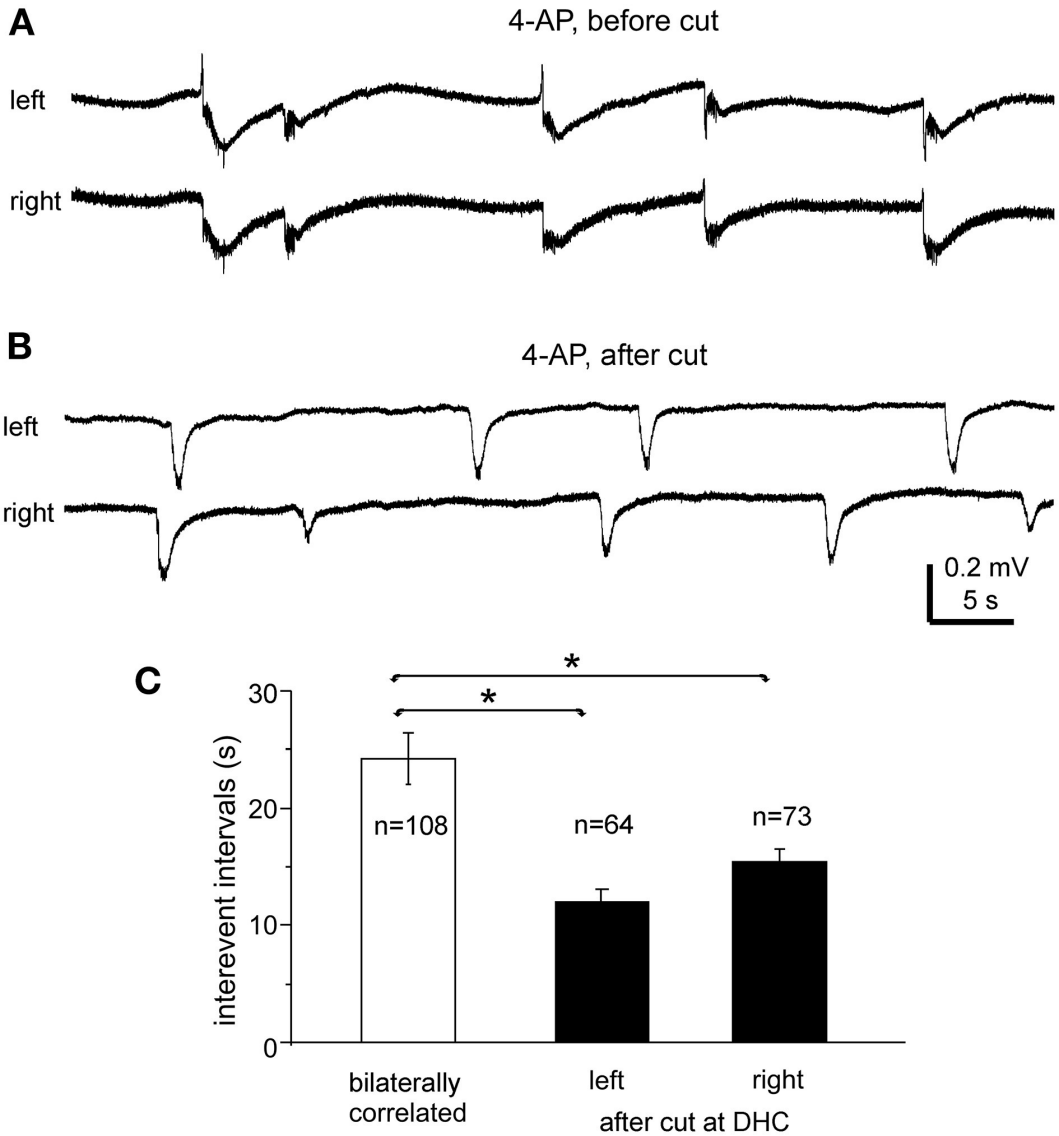
Bilateral interictal potentials observed before and following a cut at DHC. **(A,B)** A bilateral hippocampal slice obtained from a 22-day-old mouse. Self-sustained interictal field potentials were induced by 100 μM 4-AP. Representative events were collected before **(A)** and following a cut at the DHC **(B)** and illustrated after a low-pass filter (200 Hz). **(C)** Interevent intervals (mean and SE) of interictal field potentials measured from bilaterally correlated events (5 slices) and from independent left and right hippocampal events after the DHC was cut. *bilaterally correlated events vs. independent left or right events, *p* < 0.05, Mann–Whitney *U* test.

Interictal-like field potentials, but not ictal discharges, were observed in 5 of the 10 slices with evoked bilateral responses mentioned above. These interictal field potentials were correlated in bilateral CA3 areas (Figures 4B, 6A). In 108 bilaterally correlate events collected from the five slices before the DHC, corresponding interictal events displayed no evident time lag on onset in 15 events (14%); left hippocampal events led (by 41–403 ms) or lagged (from 20 to 555 ms) right hippocampal events at onset in 48 (44%) or 45 events (42%), respectively. The mean onset time lag of these bilaterally correlated interictal events was 152.0 ± 10.7 ms (Figure 4C, right). The interevent intervals of bilaterally correlated interictal events were also variable, and the mean interval was 24.3 ± 2.2 s (ranging from 0.3 to 134 s, Figure 6C).

After the DHC was cut, interictal events remained detectable in isolated left and right CA3 areas; however, regional events occurred independently with mean interevent intervals of 12.2 ± 1.2 and 15.4 ± 1.1 s for left and right hippocampal events (*n* = 63 and 74 events from five slices; Figures 6B,C). The interevent intervals were significantly different between the bilaterally correlated events and the independent left or right hippocampal events (Mann–Whitney *U* test, *p* < 0.05), but not between the independent regional events (Figure 6C). Combined, the above observations suggested that bilateral interconnections through the DHC were critical in mediating bilaterally correlated CA3 population epileptiform activities.

### Observations From Bilateral Hippocampal Slices of Adult Mice

#### Evoked Responses Observed From Bilateral CA3 Areas

**Figure 7:**
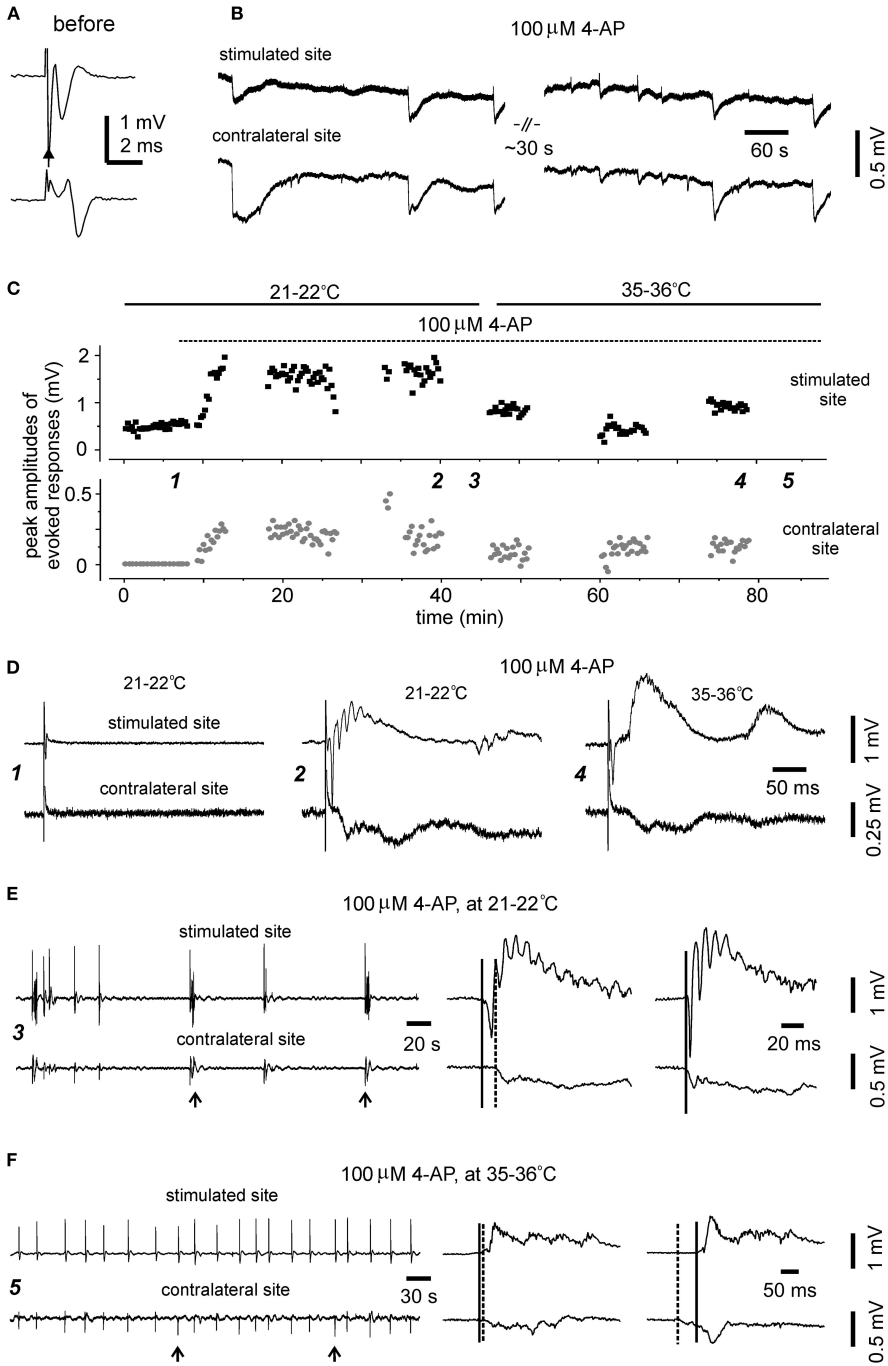
Evoked and self-sustained responses observed from bilateral hippocampal slices of adult mice. **(A,B)** A slice obtained from a 9-month-old mouse. Recordings performed at the room temperature. Bilateral responses were evoked unilaterally (filled arrow) by constant stimulations at near maximal intensity (150 μA) while the slice was perfused with standard ACSF. Averages of four consecutively evoked responses were illustrated in **(A)**. Self-sustained, epileptic form field potentials were induced by application of 100 μM 4-AP and illustrated in **(B)** after treatment with a low-pass filter (200 Hz). Note bilaterally correlated events with irregular waveforms. **(C–F)** A slice obtained from a 12-month-old mouse. Recordings performed initially at the room temperature (21–22°C) and then at 35–36°C. **(C)** Constant stimulations (150 μA) were applied unilaterally every 15 s, and peak amplitudes of evoked bilateral responses were plotted vs. time. Application of 100 μM 4-AP (dotted line) and slices recording temperatures (soled lines) are indicated above the plot. Self-sustained epileptiform filed potentials were monitored in periods without evoked responses. **(D)** Five consecutively evoked responses were collected at indicated times in **(C)** and averaged responses were presented. Note small responses of the stimulated site before 4-AP application (left); large epileptiform responses of the stimulated site and corresponding contralateral response following application of 100 μM 4-AP for about 30 min and monitored at the room temperature (middle); evoked bilateral responses collected following 4-AP application for another 30 min and monitored at 35–36°C (right). **(E)** 4-AP induced interictal field potentials were collected in the period indicated in **(C)**. Recordings were made at 21–22°C. Original data were illustrated after treatment with a band-pass filter (0.5–500 Hz). Arrowed events were expanded at right to show initial parts of corresponding bilateral events. Solid and dotted lines indicate putative onset times of interictal events of the stimulated and contralateral sites. **(F)** 4-AP induced interictal field potentials were collected in the period indicated in **(C)** (≥30 min of monitoring at 35–36°C). Illustration was similarly arranged as in **(E)**.

The preparation of bilateral hippocampal slices was carried out in 11 adult mice (9–12-month-old). Standard horizontal slices of 0.4 mm thickness were obtained from same mice and recorded to control general complications in brain dissection and vibratome sectioning procedures. Slices with visually appreciable preservation of bilateral hippocampal tissues and the connecting DHC were obtained from 7/11 adult mice (one slice per mouse). Major complications in slice preparation procedures were not evident in these 7 experiments as evoked hippocampal responses were readily observed from standard slices of same mice. However, while being perfused with standard ACSF at the room temperature (21–22°C), unilaterally elicited and bilaterally recorded CA3 responses were observed only from one of seven slices examined (Figure 7A). In remaining six slices, evoked responses were observed from the stimulated site but not contralateral site in three slices (Figure 7D, left); evoked responses were not observed from both sites in other three slices. The evoked responses observed from the stimulated site were variable, with peak amplitudes ranging from 0.1 to 1.3 mV (*n* = 4 slices).

In the three slices with evoked responses observable from the stimulated site only during standard ACSF perfusion, evoked bilateral epileptiform responses were observed from two slices following application of 100 μM 4-AP. These epileptiform responses were relatively stable while being evoked intermittently at the room temperature over a 30-min period (Figures 7C,D, middle). Evoked bilateral epileptiform responses were also observed while being monitored at 35–36°C for another 30 min (Figures 7C,D, right), although with decreased amplitude and altered waveforms relative to those evoked at the room temperature (Figures 7C,D, middle).

#### Bilaterally Correlated Epileptiform Field Potentials

Of the seven slices with visually appreciable preservation of bilateral hippocampal tissues and the connecting DHC, self-sustained epileptiform field potentials following application of 100 μM 4-AP were observed from the three slices with evoked bilateral responses but not from other four slices. These observed epileptiform potentials were bilaterally correlated and showed irregular waveforms and occurrence in one slice (Figure 7B) or intermittent interictal-like events in other two slices (Figure 7E). Ictal discharges like those observed from slices of young mice were not observed from these slices. The interictal events were relatively stable while being monitored at 35–36°C and appeared to be more periodic than those observed at 21–22°C (Figure 7F). The mean interevent interval of interictal events at 35–36°C was 25.1 ± 1.5 second (ranging from 4 to 49 s, 59 events from two slices). Corresponding bilateral interictal events occurred with the events of the stimulated site led or lagged contralateral events by 10–80 ms at onset (Figures 7E,F).

Together these observations and the above observations from slices of 28–32-day-old mice suggest that it is probable but highly variable to preserve bilateral hippocampal connections in slices of more developed or adult mice. The lack of evoked responses from the contralateral site in some slices might be partly due to functional suppression, particularly while slices were monitored at 35–36°C, as evoked bilateral responses were observed following the application of 4-AP. We speculate that 4-AP may facilitate transmitter release (Kasatkina, 2016) and improve axonal conductivity (Leussink et al., 2018) therefore enhancing synaptic signals and their spreading from the stimulated site to the contralateral site via the DHC.

## Discussion

In this study, we describe a slice preparation that may be suitable for the examination of bilateral hippocampal epileptiform activities in young mice. Our experiments showed that 600-μm-thick slices could be obtained by routine vibratome sectioning. Bilateral dorsal hippocampal and connecting DHC tissues were preserved in the slice and were largely separated from adjacent cortical/striatal tissues by ventricle spaces. As such, it was relatively easy to isolate bilateral dorsal hippocampal and DHC tissues. Bilateral CA3 areas were functionally connected in the slice, as unilateral stimulations evoked population spikes in the contralateral CA3 area. These contralateral spikes were attenuated by inhibiting synaptic transmission with cobalt and absent when a cut was made at the DHC. Self-sustained, bilaterally correlated epileptiform field potentials were observed from the bilateral CA3 areas following 4-AP application. The onsets of these epileptiform potentials varied, and the events of the stimulated site concurred with, or led or lagged contralateral events at onset. The bilaterally correlated epileptiform field potentials were dependent on bilateral interconnections through the DHC as epileptiform potentials became independent in isolated left and right CA3 areas after the DHC was cut. Together, these observations suggest that complex mechanisms may underlie initiation and correlation of population epileptiform activities in bilaterally connected CA3 circuitries *in vitro*.

Several issues remain to be further examined regarding the bilateral hippocampal slice preparation. We prepared slices from young mice (21–24 days old) in most experiments as brain tissues of young animals are generally better preserved *in vitro*, which provides greater probability of preserving brain network connectivity and examining population epileptiform activities compared to brain tissues of adult animals. We therefore used young mice as a proof-of-principle approach to explore the feasibility of this slice preparation. However, rat hippocampal neurons have been found to undergo further structural and functional alterations after the third postnatal week (Blair et al., 2013), and similar alterations may also occur in mouse hippocampal neurons. It is, therefore, of great interest to test whether the bilateral hippocampal slice is feasible for use in more mature or adult mice. We prepared bilateral hippocampal slices from adult mice (9–12 months old) to explore this issue. Evoked bilateral responses and bilaterally correlated epileptiform field potentials were observed in some of these slices, but they were inconsistent in occurrence and more variable in waveforms compared to those observed from bilateral hippocampal slices of young mice. In addition, 4-AP induced ictal discharges were not observed from bilateral hippocampal slices of adult mice. While our present observations suggest that it is probable to preserve bilateral hippocampal connections in slices of adult mice, further works are required to validate the feasibility and reliability of this approach for its employment in adult mice.

Commonly used or standard transverse hippocampal slices have thickness of 300–400 μm and a narrow strip of CA3 and CA1 pyramidal neuronal axons (stratum alveus). Relative to the standard slices, the bilateral hippocampal slices used in our present experiments were thicker (600 μm) and encompassed a larger area of pyramidal neuronal axon layer, which may limit slice oxygenation while being recorded *in vitro*. We therefore recorded the bilateral hippocampal slices at a room temperature (21–22°C) in most experiments to reduce slice oxygen demand and to better preserve bilateral hippocampal connectivity *in vitro*. To explore potential temperature influences on bilateral hippocampal activities, we conducted some experiments in which the bilateral hippocampal slices of young or adult mice were monitored initially at the room temperature and then at 35–36°C. Evoked bilateral hippocampal responses and bilaterally correlated epileptiform field potentials were detectable at 35–36°C but with decreased amplitudes and/or altered waveforms in comparison to those observed at the room temperature. While remain to be verified, these observations suggest that the DHC-mediated bilateral hippocampal connectivity can be at least partly retained at more physiological temperatures. As the use of near physiological temperatures generally decreases the survival time of acutely prepared brain slices (Dulla et al., 2018), structural and/or functional deterioration of bilateral hippocampal tissues particularly the connecting DHC at 35–36°C may largely account for the diminished and/variable bilateral hippocampal activities observed. Further works are necessary to improve our experimental conditions and to achieve better preservation of the bilateral hippocampal connectivity at more physiological temperatures.

Unlike the hippocampal commissure in primates and humans (Lamantia and Rakic, 1990; Gloor et al., 1993), the VHC is more prominent than the DHC in rodents. Durand and co-workers developed a bilateral ventral hippocampal slice and demonstrated the significance of the VHC in the synchronization of bilateral hippocampal epileptiform activities (Toprani and Durand, 2013a,b; Wang et al., 2014). Further work is needed to explore the feasibility of obtaining bilateral ventral hippocampal slices from young and adult mice as per these previous studies. Despite the above-mentioned weaknesses and limitations, our results may help future *in vitro* investigations of bilateral hippocampal activities in mouse models.

## Data Availability Statement

The raw data supporting the conclusions of this article will be made available by the authors, without undue reservation.

## Ethics Statement

The animal study was reviewed and approved by the Animal Care Committee of the University Health Network in accordance with the Guidelines of the Canadian Council on Animal Care.

## Author Contributions

HL performed experiments. HL and LZ conducted data analysis. LZ and PC wrote manuscript. All authors participated in literature search, experimental design, and data discussion.

## Conflict of Interest

The authors declare that the research was conducted in the absence of any commercial or financial relationships that could be construed as a potential conflict of interest.
